# Preconception Management of Hyperthyroidism and Thyroid Status in Subsequent Pregnancy: A Population-Based Cohort Study

**DOI:** 10.1210/clinem/dgad276

**Published:** 2023-05-18

**Authors:** Caroline Minassian, Lowri A Allen, Onyebuchi Okosieme, Bijay Vaidya, Peter Taylor

**Affiliations:** Faculty of Epidemiology and Population Health, Department of Non-communicable Disease Epidemiology, London School of Hygiene and Tropical Medicine, London WC1E 7HT, UK; Diabetes Research Group, Cardiff University School of Medicine, Cardiff CF14 4XN, UK; Thyroid Research Group, Systems Immunity Research Institute Medicine, Cardiff University School of Medicine, Cardiff CF14 4XN, UK; Department of Endocrinology, Royal Devon & Exeter Hospital, University of Exeter Medical School, Exeter EX2 5DW, UK; Thyroid Research Group, Systems Immunity Research Institute Medicine, Cardiff University School of Medicine, Cardiff CF14 4XN, UK

**Keywords:** pregnancy, thyroid function, hyperthyroidism, TSH, FT3, FT4, thyroid stimulating hormone, thyroxine, tri-iodothyronine, CPRD, PTU, carbimazole

## Abstract

**Context:**

Optimal thyroid status in pregnancy is essential in reducing the risk of adverse outcomes. The management of hyperthyroidism in women of reproductive age poses unique challenges and it is unclear how preconception treatment strategies impact on thyroid status in subsequent pregnancy.

**Objective:**

We aimed to determine trends in the management of hyperthyroidism before and during pregnancy and to assess the impact of different preconception treatment strategies on maternal thyroid status.

**Methods:**

We utilized the Clinical Practice Research Datalink database to evaluate all females aged 15-45 years with a clinical diagnosis of hyperthyroidism and a subsequent pregnancy (January 2000 to December 2017). We compared thyroid status in pregnancy according to preconception treatment, namely, (1) antithyroid drugs up to or beyond pregnancy onset, (2) definitive treatment with thyroidectomy or radioiodine before pregnancy, and (3) no treatment at pregnancy onset.

**Results:**

Our study cohort comprised 4712 pregnancies. Thyrotropin (TSH) was measured in only 53.1% of pregnancies, of which 28.1% showed suboptimal thyroid status (TSH >4.0 mU/L or TSH <0.1 mU/L plus FT4 >reference range). Pregnancies with prior definitive treatment were more likely to have suboptimal thyroid status compared with pregnancies starting during antithyroid drug treatment (odds ratio 4.72, 95% CI 3.50-6.36). A steady decline in the use of definitive treatment before pregnancy was observed from 2000 to 2017. One-third (32.6%) of first trimester carbimazole-exposed pregnancies were switched to propylthiouracil while 6.0% of propylthiouracil-exposed pregnancies switched to carbimazole.

**Conclusion:**

The management of women with hyperthyroidism who become pregnant is suboptimal, particularly in those with preconception definitive treatment, and needs urgent improvement. Better thyroid monitoring and prenatal counseling are needed to optimize thyroid status, reduce teratogenic drug exposure, and ultimately reduce the risk of adverse pregnancy outcomes.

Thyroid hormones are essential for maintaining pregnancy and offspring development (1). Hyperthyroidism is common in women of child-bearing age (2, 3), although pathological hyperthyroidism is relatively rare in pregnancy with an approximate prevalence rate in Western countries of 0.5% to 1.3% for established or pre-existing Graves disease, 0.05% for new-onset Graves disease, and 0.1% for a toxic thyroid nodule (4, 5). Uncontrolled hyperthyroidism has adverse consequences in pregnancy, including increased risks of pre-eclampsia, preterm delivery, low birthweight, and fetal loss (1). It is also increasingly recognized that even modest variation in thyroid status within and just outside the gestational reference range is associated with important obstetric and offspring outcomes (6–8).

The management of women who are thyrotoxic during pregnancy is challenging, as both hyperthyroidism and antithyroid drugs are associated with adverse outcomes (1, 3, 9). Carbimazole (CBZ) and methimazole (MMI) are associated with significant congenital malformations including aplasia cutis, esophageal atresia, omphalocele, and omphalomesenteric duct abnormalities (10). The use of propylthiouracil (PTU) is also associated with maternal liver injury (11) and increased risk of birth defects including hydronephrosis (12–15), although the birth defects are thought to be milder and less common than with CBZ/MMI. As a result PTU is the preferred antithyroid agent in the first trimester (3).

A strategy to reduce the risk of birth defects and maternal liver injury was proposed by utilizing PTU in the first trimester and switching to CBZ/MMI after the first trimester (16). More recently, large studies from Denmark (14) and Korea (15) have raised concerns about switching in pregnancy as exposure to both antithyroid drugs appears to result in more adverse outcomes. Two recent meta-analyses have suggested that the offspring of women who switched antithyroid drugs in pregnancy had high risks of congenital anomalies that were even greater than that of offspring of women with single-drug exposure (17, 18). Additionally, concern has been raised due to a deterioration in thyroid status that may result from a switch in antithyroid drug. Another approach for women who are established on antithyroid drugs at conception is to stop antithyroid drug treatment as soon as pregnancy is confirmed, with very close monitoring of thyroid status in the expectation that hyperthyroidism is unlikely to recur during pregnancy (19). However, this approach is only possible in women established on stable low doses of antithyroid drugs for several months (3, 16, 20).

Given these challenges, many endocrinologists offer definitive treatment with radioactive iodine or surgery to women with hyperthyroidism who plan to get pregnant in the future. This reduces risks of Graves disease relapse or exposure to antithyroid drugs in the ensuing pregnancy. However, these women will usually require thyroid hormone replacement in the form of levothyroxine prior to pregnancy. We have previously shown using UK primary care data that most women with hypothyroidism established on levothyroxine prior to pregnancy have suboptimal control during pregnancy, which is associated with increased odds of fetal loss (21). While the majority of patients with treated hypothyroidism have autoimmune Hashimoto thyroiditis (2), this small proportion of patients with prior definitive treatment for hyperthyroidism may be particularly vulnerable to hypothyroidism in pregnancy due to extremely limited thyroid reserve and may therefore fail to meet the increased thyroid demands of pregnancy (1).

However, there are limited data on the impact of these various preconception strategies on thyroid status in subsequent pregnancy, and it is unclear how much this matter is discussed when counseling women who are offered definitive treatment. We therefore sought to determine trends in the management of hyperthyroidism before and during pregnancy and to explore the impact of different management strategies in women with pre-existing hyperthyroidism on maintaining optimal thyroid status during pregnancy using a national UK clinical research database.

## Materials and Methods

### Study Design and Data Sources

We conducted a cohort study to determine trends in the management of hyperthyroidism before and during pregnancy, and to compare women's thyroid status in pregnancy according to the type of prior treatment for hyperthyroidism they received, using data from the Clinical Practice Research Datalink (CPRD) GOLD database, the CPRD-GOLD Pregnancy Register, and linked hospitalization data.

CPRD-GOLD is a database of anonymized primary care health records for patients across the UK (22), capturing demographic characteristics, clinical diagnoses and procedures, primary care prescribing, laboratory tests, and referrals to hospital and specialist care. Data validity has been shown to be high (23). The January 2018 database build from which our study population was selected included data for over 15 million patients, of whom 6.15 million were currently registered.

The CPRD-GOLD Pregnancy Register is generated monthly using a validated algorithm, combining information across the primary care record relating to antenatal scans and appointments, expected delivery dates, and pregnancy outcomes, to date and characterize pregnancies (24). Live-birth deliveries are linked to records of infants registered at the same practice as their mother, by the CPRD mother–baby linkage algorithm (25).

Linkage to other health-related patient datasets, including Hospital Episode Statistics (HES) and Index of Multiple Deprivation (IMD) data, are available for 75% of patients registered with CPRD-GOLD in England based on practice-level consent. Linked HES inpatient and outpatient data were used to maximize ascertainment of definitive treatment for hyperthyroidism. Patient postcode–linked measures of IMD (2010) were used to measure social deprivation.

### Study Population and Observation Period

Our study population comprised all females aged 15-45 years registered with a CPRD-GOLD practice, with a clinical diagnosis of hyperthyroidism (see Table S1 for read codes (26)), and a subsequent pregnancy recorded in the Pregnancy Register during the period from January 1 2000, to December 31, 2017. To be included in the study, patients were required to have been registered at the GP practice for at least 1 year before the start of pregnancy to ensure that covariates were adequately captured, and to enable the temporal sequence of hyperthyroidism diagnosis, its treatment, and pregnancy to be determined.

Patients were followed up from the latest of 1 year after registering with the practice, their earliest hyperthyroidism diagnosis, their 15th birthday, the practice meeting CPRD quality standards, and 1 January 2000. Follow-up ended at the earliest of their 46th birthday, leaving the practice, last data collection from the practice, death, or the study end date (31 December 2017). Pregnancies starting and ending during follow-up were identified from the Pregnancy Register. We restricted to pregnancies with known outcomes (delivery or early pregnancy loss) due to uncertainty regarding the start, end, and trimester dates of pregnancies whose outcomes were unknown. Pregnancies whose dates appeared to overlap with another pregnancy episode were excluded due to uncertainty regarding their timing and outcome (27), except for deliveries with a linked infant (identified using the CPRD-GOLD Mother–Baby linkage algorithm) for which the outcome and timing could be more reliably ascertained.

All pregnancies meeting the above eligibility criteria were included in the descriptive analysis of trends in hyperthyroidism management. The main analysis, comparing thyroid status during pregnancy by prior treatment for hyperthyroidism, was restricted to the subset of pregnancies with a thyrotropin (TSH) test record.

### Exposure

The exposure of interest for the main analysis was the treatment received for hyperthyroidism before pregnancy. Records of definitive treatment with radioiodine and thyroid surgery (subtotal and total thyroidectomy) were identified from primary care data using Read codes (version 2) (28), and from linked HES data using International Classification of Diseases ICD-10 codes, and Classification of Interventions and Procedures OPCS-4 codes.

Antithyroid drug prescriptions were identified from primary care data using a list of antithyroid drug codes that we compiled by searching the CPRD-GOLD product dictionary on drug substance name, product name, British National Formulary chapter, and British National Formulary codes. For each antithyroid drug type (PTU or CBZ), we determined prescription length from the number of treatment days (when recorded), or else by dividing the total quantity of the drug prescribed by the daily dosage. When daily dose was missing, the mode daily dose for records of the same drug was used. To determine continuous coverage periods, successive prescriptions for the same drug type were appended, allowing up to 30 days of lag time (the median antithyroid prescription duration) between prescriptions. When a CBZ prescription overlapped with a PTU prescription, we assumed that the patient had switched to the later-prescribed drug and vice versa.

We classified pregnancies into 3 exposure groups: (1) taking antithyroid drugs at the start of pregnancy (either PTU or CBZ), based on a prescription issued before pregnancy, with coverage ending on or after the pregnancy start date; (2) prior definitive treatment with radioiodine or thyroid surgery; and (3) no prior definitive treatment and not taking antithyroid drugs at the start of pregnancy. Pregnancies meeting criteria for both exposure groups 1 and 2 were included in group 2, as this allowed us to assess the effect of definitive treatment on thyroid status in pregnancy, regardless of its effectiveness in treating thyrotoxicosis before pregnancy.

For the descriptive analysis of trends in hyperthyroidism management, we assessed treatments received before and during pregnancy (type of antithyroid drug, radioiodine, and thyroid surgery).

### Outcome

The primary outcome was suboptimal thyroid status during pregnancy. This was ascertained from thyroid test results in pregnancy indicating TSH and free thyroxine (FT4) levels, recorded in primary care data using Test Entity Types 203 and 345, respectively. Secondary outcomes were suboptimal thyroid status during the first trimester, and during the second and/or third trimesters.

Consistent with guideline recommendations for targeting TSH levels in pregnant women with thyroid disease, we considered optimal TSH levels to fall within the range 0.1 to 4.0 mU/L (16). However, because TSH levels may remain suppressed for several months after starting treatment for thyrotoxicosis (29), pregnancies with low TSH only (ie, normal FT4 [within the recorded reference range], or FT4 data not available) were considered normal. Thus, we defined suboptimal thyroid status as (1) TSH >4.0 mU/L, or (2) TSH <0.1 mU/L in the presence of raised FT4 (above the reference range). For women with multiple TSH or FT4 tests during the relevant time period of analysis (ie, throughout pregnancy or within specific trimesters), any TSH or FT4 level outside the reference range was used to determine thyroid status.

### Covariates

Maternal factors considered as potential confounders were age at pregnancy start, year of pregnancy start, deprivation, body mass index (BMI), smoking status, multiple pregnancy, and prepregnancy diabetes and hypertension. Deprivation was measured by IMD (in quintiles, with higher values indicating greater deprivation), based on patient postcode when available, supplemented with practice-level measures. BMI (calculated from weight and height, kg/m^2^) was categorized as underweight <18.5, healthy weight 18.5 to 24.9, overweight 25 to 29.9, and obese ≥30. We used the BMI measure closest to the start of pregnancy allowing for measures throughout the first trimester. We defined smoking status from records during pregnancy (when available) or the most recent record up to 10 years before pregnancy. Smoking status was classified as “current smoker”, “ex-smoker,” or “nonsmoker”, with nonsmoker reclassified as ex-smoker if there was previous evidence of smoking.

Pregnancies with more than 1 fetus were identified from the Pregnancy Register, which classifies pregnancies as “multiple” based on read codes for multiple pregnancy recorded antenatally and codes for multiple birth, recorded at delivery through to 8 weeks postpartum or in linked infant records. Pre-existing diabetes and hypertension were identified by the presence of a read code for either condition before the start of pregnancy.

### Statistical Analysis

#### Descriptive analysis of included pregnancies

Covariates and exposure to hyperthyroidism treatment before and during pregnancy were tabulated for the full pregnancy cohort, the analysis cohort (pregnancies with a TSH record), and additionally for pregnancies without a TSH record. We calculated standardized differences to identify any key differences in the covariate distribution between the last 2 groups. We considered a standardized difference ≥0.2 to denote a meaningful imbalance in baseline covariates (30). The frequency of TSH and FT4 tests recorded in pregnancy and the distribution of TSH and FT4 levels were also described.

Among pregnancies exposed to antithyroid drug treatment, we identified patterns of drug switching, defined as exposure to 1 antithyroid drug in the first trimester and a subsequent prescription for the other drug during or after the first trimester, for instance, from PTU to CBZ after the first trimester, consistent with the 2012 guideline recommendations (31). We reported frequencies of any switching and switching in accordance with the guidelines.

#### Analysis of trends in hyperthyroidism management before and during pregnancy

We assessed the frequency of receipt of definitive treatment before pregnancy by calculating the proportion of pregnancies with a prior record of radioiodine or thyroid surgery, by calendar period (3-year bands from 2000 to 2017). We assessed treatment patterns for hyperthyroidism during pregnancy by calculating the proportion of pregnancies occurring during antithyroid drug treatment (CBZ, PTU, or both drugs at different stages) by calendar period.

#### Analysis of thyroid control in pregnancy by prior treatment status

Absolute risks of suboptimal thyroid status in pregnancy were calculated for each exposure group in the analysis cohort: (1) pregnancies starting during antithyroid drug treatment, (2) pregnancies exposed to prior definitive treatment, and (3) pregnancies with no prior definitive treatment and not starting during antithyroid drug treatment. To assess the association between the type of treatment received for hyperthyroidism before pregnancy and subsequent thyroid status during pregnancy, we calculated odds ratio (OR) and 95% CI for suboptimal thyroid status at any time during pregnancy and by trimester (first, second/third), comparing definitively treated and untreated groups relative to the antithyroid drug–treated group. Multivariable logistic regression was used to adjust for confounding, with random effects to account for within-person clustering because some women having more than 1 included pregnancy. A complete case analysis was used to handle missing data.

In a post hoc descriptive analysis among women with radioiodine or thyroid surgery before pregnancy, we identified levothyroxine prescriptions after treatment and classified their pregnancies as having or not having prior exposure to levothyroxine. We calculated absolute risks of suboptimal thyroid status in pregnancy separately for pregnancies with and without prior levothyroxine exposure. We conducted an additional post hoc analysis to assess preconception TSH levels and to what extent suboptimal thyroid status in early pregnancy may reflect inadequate thyroxine replacement in the months leading up to conception. We identified TSH records in the 90 days before conception and calculated the proportion of pregnancies with TSH levels >2.5 mU/L before conception overall and by treatment group. Among pregnancies with prior radioiodine or thyroid surgery, we calculated absolute risks of suboptimal thyroid status in the first trimester separately for those with and without preconception TSH >2.5 mU/L.

The following sensitivity analyses were performed, restricting to (1) pregnancies with FT4 data (a FT4 record and corresponding reference range) to address possible misclassification of thyroid status in pregnancies with suppressed TSH and missing FT4 data; and (2) women eligible for HES linkage, for whom radioiodine and thyroid surgery may be captured more fully. Finally, we repeated the primary analysis using a lower TSH threshold of 2.5 mU/L (instead of 4 mU/L) to determine thyroid status during pregnancy to address possible under-ascertainment of suboptimal control. Thus, we defined suboptimal thyroid status as (1) TSH >2.5 mU/L, or (2) TSH <0.1 mU/L in the presence of raised FT4 (above the reference range).

All analyses were conducted using STATA (StataCorp LP, version 17.0).

### Ethics

Ethics approval was obtained from the CPRD Independent Scientific Advisory Committee (approval number 18_093) and the London School of Hygiene and Tropical Medicine Research Ethics Committee (approval number 28355).

## Results

### Description of Full Cohort

**Figure 1.**
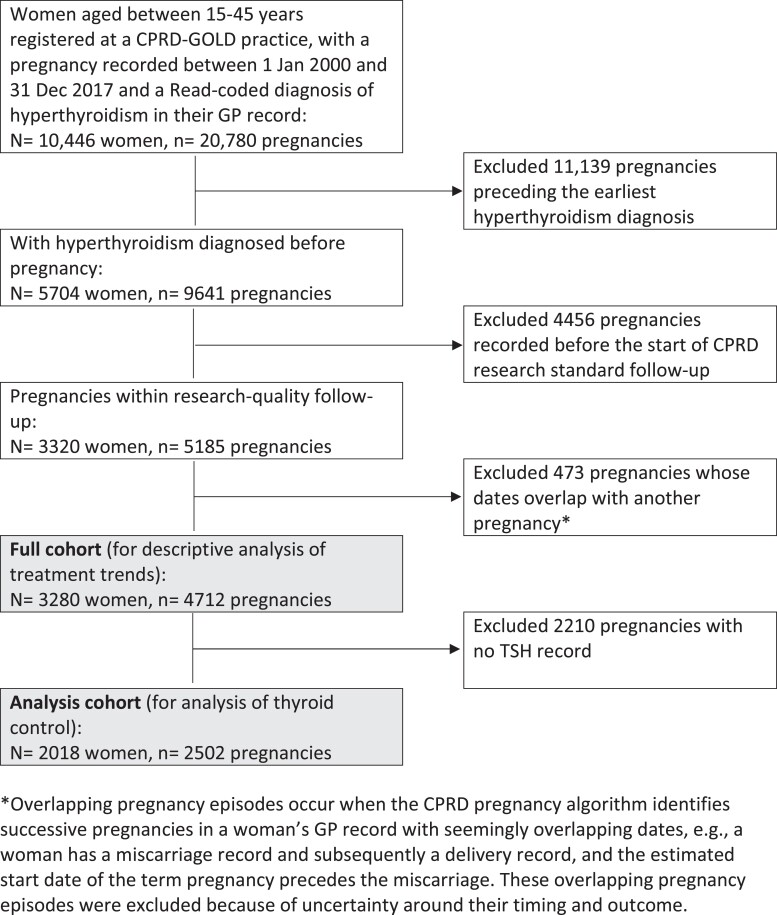
Identification of study cohorts.

Identification of our study cohorts is shown in Fig. 1. Our full study cohort comprised 4712 pregnancies among 3280 women, with median age at conception of 33 years, interquartile range (IQR) 29-36 years (Table S2 (26)). Overall, 2502 pregnancies (53.1%) had a TSH record (median 2 records, IQR 1-3), 1832 pregnancies (38.9%) had a FT4 record (median 1 record, IQR 1-3), of which 1454 (79.4%) had a FT4 reference range, and 1804 pregnancies had both a TSH and FT4 record (72.1% of pregnancies with a TSH record). Women treated with radioiodine or thyroid surgery before pregnancy were less likely to have TSH or FT4 recorded during pregnancy than women who became pregnant on antithyroid drugs (65.3% vs 73.8% had TSH recorded, 49.8% vs 61.3% had FT4 recorded). The distribution of TSH and FT4 tests and levels in pregnancy are shown in Tables 1 and 2.

**Table 1.** TSH test frequency and levels by treatment status at start of pregnancy

	Any treatment status	Taking ATDs	Prior definitive treatment	No prior definitive treatment and not taking ATDs
All pregnancies	4712 (100)	615 (100)	832 (100)	3265 (100)
Pregnancies with a TSH record	2502 (53.1)	454 (73.8)	543 (65.3)	1505 (46.1)
Pregnancies with a TSH record in first trimester	1858 (39.4)	376 (61.1)	412 (49.5)	1070 (32.8)
Pregnancies with a TSH record in second or third trimester	1616 (34.3)	291 (47.3)	360 (43.3)	965 (29.6)
Number of TSH records in pregnancy*^a^*				
1	1125 (45.0)	162 (35.7)	227 (41.8)	736 (48.9)
2	560 (22.4)	103 (22.7)	115 (21.2)	342 (22.7)
3	348 (13.9)	65 (14.3)	85 (15.7)	198 (13.2)
≥4	469 (18.7)	124 (27.3)	116 (21.4)	229 (15.2)
Median (IQR)	2 (1-3)	2 (1-4)	2 (1-3)	2 (1-3)
TSH level in pregnancy (mU/L) median (IQR)				
Any time in pregnancy	1.14 (0.31-2.50)	0.67 (0.05-1.49)	2.20 (0.66-5.22)	1.12 (0.40-2.30)
First trimester	1.22 (0.25-3.07)	0.50 (0.03-1.55)	2.97 (0.93-7.07)	1.20 (0.37-2.69)
Second or third trimester	1.09 (0.35-2.21)	0.79 (0.10-1.43)	1.69 (0.50-4.17)	1.07 (0.44-2.10)

Data are presented as n (%), unless stated otherwise.

n (% of pregnancies with a TSH record).

**Table 2.** FT4 test frequency and levels by treatment status at start of pregnancy

	Any treatment status	Taking ATDs	Prior definitive treatment	No prior definitive treatment and not taking ATDs
All pregnancies	4712 (100)	615 (100)	832 (100)	3265 (100)
Pregnancies with a FT4 record	1832 (38.9)	377 (61.3)	414 (49.8)	1041 (31.9)
Pregnancies with a FT4 record in first trimester	1293 (27.4)	293 (47.6)	296 (35.6)	704 (21.6)
Pregnancies with a FT4 record in second or third trimester	1134 (24.1)	224 (36.4)	268 (32.2)	642 (19.7)
Number of FT4 records in pregnancy*^a^*				
1	930 (50.8)	166 (44.0)	203 (49.0)	561 (53.9)
2	389 (21.2)	74 (19.6)	85 (20.5)	230 (22.1)
3	243 (13.3)	56 (14.9)	63 (15.2)	124 (11.9)
≥4	270 (14.7)	81 (21.5)	63 (15.2)	126 (12.1)
Median (IQR)	1 (1-3)	2 (1-3)	2 (1-3)	1 (1-2)
FT4 level in pregnancy (pmol/L) median (IQR)				
Any time in pregnancy	14.3 (12.1-17.2)	13.7 (11.8-16.9)	15.6 (13.1-18.3)	14.1 (12.1-17.0)
First trimester	15.6 (13.0-19.0)	15.0 (12.5-19.3)	16.2 (13.7-19.0)	15.5 (13.5-18.8)
Second or third trimester	13.6 (11.8-16.0)	13.0 (11.4-15.0)	15.0 (13.0-17.5)	13.3 (11.5-15.2)

Data are presented as n (%), unless stated otherwise.

Abbreviations: ATD, antithyroid drug; FT4, free thyroxine; IQR, interquartile range; TSH, thyrotropin.

^*a*^n (% of pregnancies with a FT4 record).

### Hyperthyroidism Management Before and During Pregnancy

Of the 4712 pregnancies in our full cohort, 174 pregnancies (3.7%) occurred after radioiodine, 658 (14.0%) after thyroid surgery, and 9 (0.2%) after both types of treatment (Table S2 (26)). Radioiodine or thyroid surgery during pregnancy were extremely uncommon, occurring in just 7 pregnancies (0.1%).

**Figure 2.**
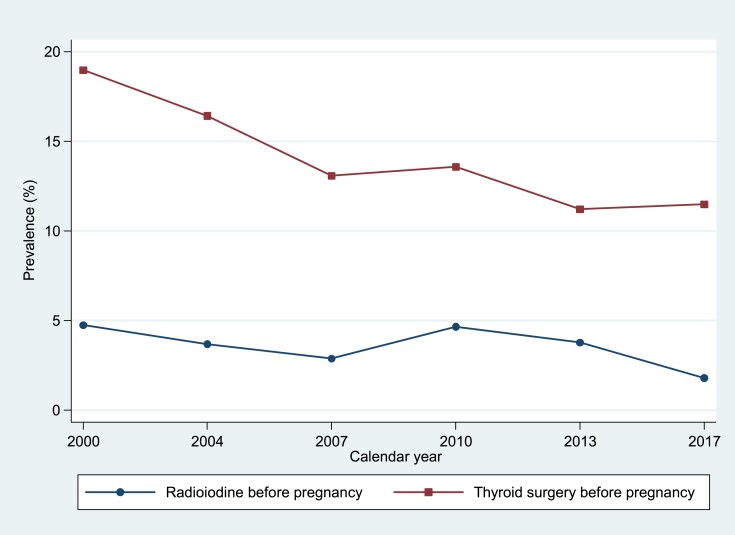
Prevalence of radioiodine and thyroid surgery before pregnancy 2000-2017.

Between 2000 and 2008, the prevalence of radioiodine before pregnancy fell from 4.7% to 2.9%, returning to 4.7% by 2011, then dropping further to 1.8% by 2017. The prevalence of thyroid surgery before pregnancy decreased more steadily over the study period, from 19.0% to 11.5% (2000-2017) (Fig. 2).

**Figure 3.**
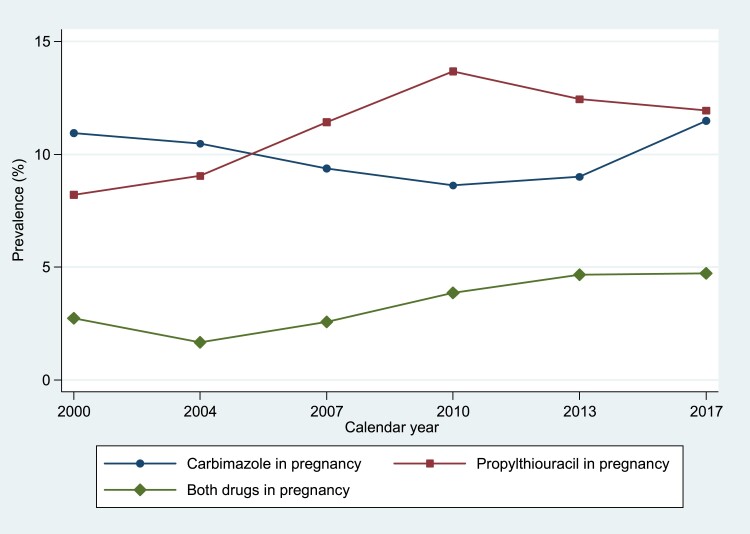
Prevalence of antithyroid drug treatment during pregnancy 2000-2017.

Overall, 837 pregnancies (17.8%) were exposed to antithyroid drug treatment: 302 to CBZ only (6.4%), 379 to PTU only (8.0%), and 156 (3.3%) to both drugs at different stages (Table S2 (26)). Between 2000 and 2011, the proportion of pregnancies prescribed CBZ fell from 10.9% to 8.6%, then gradually increased to 11.5% by 2017. A reverse pattern was seen for PTU, with a steady increase in prescribing from 8.2% to 13.7% (2000-2011), followed by a decrease to 11.9% by 2017. The proportion of pregnancies prescribed both drugs decreased from 2.7% in 2000 to 1.7% in 2004, increasing thereafter to 4.7% by the end of the study period (Fig. 3).

Switching antithyroid drug type occurred in 155 pregnancies, affecting 19.8% of first trimester antithyroid drug–exposed pregnancies, with a higher proportion switching from CBZ to PTU (32.6% of first trimester CBZ-exposed pregnancies) than from PTU to CBZ (6.0% of first trimester PTU-exposed pregnancies) (Table 3). Most PTU to CBZ switches (n = 22, 78.6%) occurred after the first trimester compared with just 23.0% of CBZ to PTU switches (n = 32).

**Table 3.** Patterns of antithyroid drug use during pregnancy among the full cohort

	Pregnancies exposed to antithyroid drug treatment n (column %)	Switching*^a^* antithyroid drug n (row %)
Total	837 (100)	155*^b^* (18.5)
First trimester exposure to any antithyroid drug	785 (93.8)	155*^b^* (19.8)
First trimester exposure to PTU	470 (56.2)	28 (6.0)
First trimester exposure to CBZ	426 (50.9)	139 (32.6)

Abbreviations: CBZ, carbimazole; PTU, propylthiouracil

^*a*^Switching defined as exposure to 1 type of antithyroid drug (PTU or (CBZ)) in the first trimester and a subsequent prescription for the other drug (during or after the first trimester).

^*b*^Includes 12 pregnancies in which both types of switching occurred (PTU to CBZ and CBZ to PTU).

### Description of Analysis Cohort

Pregnancies with no TSH record (n = 2210, 46.9% of the full cohort) were excluded from the main analysis. Pregnancies without a TSH record were less likely than pregnancies with a TSH record to have had prior radioiodine or thyroid surgery or to be exposed to antithyroid drugs at conception (standardized difference = 0.45) and were more likely to end in early pregnancy loss (standardized difference = 0.60) (Table S3 (26)). There was a small difference in IMD between pregnancies with and without a TSH record (standardized difference = 0.18), with higher deprivation among excluded pregnancies; however, there were no marked differences in the distribution for all other covariates.

Our analysis cohort comprised 2502 pregnancies with a TSH record, among 2018 women, of whom 1609 (79.7%) had 1 included pregnancy, 341 (16.9%) had 2, and 68 (3.4%) had 3 or more.

Pregnancies were categorized into 3 exposure groups: 454 pregnancies (18.1%) started during antithyroid drug treatment, 543 (21.7%) had prior treatment with radioiodine or thyroid surgery, and 1505 (60.2%) had no prior radioiodine or thyroid surgery and were not exposed to antithyroid drugs at the start (Table 4). Of this latter group, 10.1% were subsequently exposed to antithyroid drug treatment during pregnancy.

**Table 4.** Baseline characteristics of the analysis cohort stratified by treatment status at start of pregnancy

	Total n = 2502 pregnancies	Taking antithyroid drugs n = 454 pregnancies	Prior definitive treatment n = 543	No prior definitive treatment and not taking antithyroid drugs n = 1505 pregnancies
Maternal age (years)				
<24	210 (8.4)	56 (12.3)	29 (5.3)	125 (8.3)
25-29	549 (21.9)	102 (22.5)	127 (23.4)	320 (21.3)
30-34	919 (36.7)	160 (35.2)	198 (36.5)	561 (37.3)
35+	824 (32.9)	136 (30)	189 (34.8)	499 (33.2)
Median (IQR)	33 (29-36)	32 (28-36)	33 (29-36)	33 (29-36)
Calendar year of pregnancy				
2000-2002	209 (8.4)	34 (7.5)	66 (12.2)	109 (7.2)
2003-2005	462 (18.5)	84 (18.5)	108 (19.9)	270 (17.9)
2006-2008	548 (21.9)	108 (23.8)	105 (19.3)	335 (22.3)
2009-2011	562 (22.5)	105 (23.1)	128 (23.6)	329 (21.9)
2012-2014	480 (19.2)	81 (17.8)	96 (17.7)	303 (20.1)
2015-2017	241 (9.6)	42 (9.3)	40 (7.4)	159 (10.6)
Pregnancy outcome				
Delivery	2109 (84.3)	374 (82.4)	451 (83.1)	1284 (85.3)
Pregnancy loss	393 (15.7)	80 (17.6)	92 (16.9)	221 (14.7)
Multiple pregnancy	25 (1)	<5	<5	21 (1.4)
Index of multiple deprivation quintile				
1 (least deprived)	578 (23.1)	108 (23.8)	110 (20.3)	360 (23.9)
2	457 (18.3)	90 (19.8)	97 (17.9)	270 (17.9)
3	507 (20.3)	86 (18.9)	116 (21.4)	305 (20.3)
4	514 (20.5)	98 (21.6)	113 (20.8)	303 (20.1)
5 (most deprived)	446 (17.8)	72 (15.9)	107 (19.7)	267 (17.7)
Body mass index (kg/m^2^)				
<18.5 underweight	68 (2.7)	17 (3.7)	6 (1.1)	45 (3)
18.5-24.9 normal	1263 (50.5)	245 (54)	259 (47.7)	759 (50.4)
25-29.9 overweight	632 (25.3)	115 (25.3)	140 (25.8)	377 (25)
30+ obese	407 (16.3)	51 (11.2)	108 (19.9)	248 (16.5)
Missing	132 (5.3)	26 (5.7)	30 (5.5)	76 (5)
Median (IQR)	24 (22-28)	24 (22-27)	25 (22-29)	24 (22-28)
Smoking status				
Nonsmoker	1229 (49.1)	222 (48.9)	236 (43.5)	771 (51.2)
Current smoker	488 (19.5)	94 (20.7)	125 (23)	269 (17.9)
Exsmoker	784 (31.3)	137 (30.2)	182 (33.5)	465 (30.9)
Missing	1 (0)	1 (0.2)	0 (0)	0 (0)
Pre-existing diabetes	393 (15.7)	90 (19.8)	77 (14.2)	226 (15)
Pre-existing hypertension	82 (3.3)	9 (2)	24 (4.4)	49 (3.3)

Abbreviation: IQR, interquartile range.

Data are presented as n (%), unless stated otherwise.

The median age at conception was 33 years (IQR 29-36 years) (Table 4). Approximately half of women had normal prepregnancy weight (n = 1263 pregnancies, 50.5%), 41.6% were overweight or obese (n = 1039), few were underweight (n = 68, 2.7%), and 5.3% had missing BMI data (n = 132). One-fifth of women were current smokers (n = 488 pregnancies, 19.5%). Less than one-quarter of women (n = 578 pregnancies, 23.1%) were from areas ranked as the least deprived, and 17.8% (n = 446) were from the most deprived areas. Prepregnancy diabetes and hypertension affected 393 pregnancies (15.7%) and 82 pregnancies (3.3%) respectively. Most pregnancies were singletons, just 25 (1.0%) were multifetal.

### Analysis of Thyroid Control in Pregnancy by Prior Treatment Status

Overall, 702 pregnancies (28.1%) had suboptimal thyroid status, of which 570 (81.2%) were due to elevated TSH and 132 (18.8%) due to suppressed TSH plus raised FT4. The absolute risk of suboptimal thyroid status at any time in pregnancy was highest among pregnancies with prior radioiodine or thyroid surgery (51.9%) and lowest among pregnancies starting during antithyroid drug treatment (19.4%). Of pregnancies with no prior radioiodine or thyroid surgery and not exposed to antithyroid drugs at the start, 22.1% had suboptimal thyroid status (Table 5).

**Table 5.** Association between treatment status at start of pregnancy and thyroid status during pregnancy among women with hyperthyroidism in the UK, 2000-2017

Treatment status at start of pregnancy	Number of pregnancies (column %)	Number with suboptimal thyroid status (row %)	Crude OR (95% CI), *P*-value	Adjusted OR (95% CI), *P* value
**Primary analysis**	2502 (100)	702 (28.1)		
Taking antithyroid drugs	454 (18.2)	88 (19.4)	ref	ref
Prior definitive treatment	543 (21.7)	282 (51.9)	4.48 (3.31-6.06), *P* < .001	4.72 (3.50-6.36), *P* < .001
No prior definitive treatment and not taking antithyroid drugs	1505 (60.1)	332 (22.1)	1.17 (0.89-1.55), *P* = .261	1.21 (0.92-1.60), *P* = .164
**Secondary analysis of thyroid status in the first trimester**	1858 (100)	511 (27.5)		
Taking antithyroid drugs	376 (20.2)	79 (21.0)	ref	ref
Prior definitive treatment	412 (22.2)	198 (48.1)	3.47 (2.49-4.82), *P* < .001	3.66 (2.64-5.07), *P* < .001
No prior definitive treatment and not taking antithyroid drugs	1070 (57.6)	234 (21.9)	1.05 (0.77-1.42), *P* = .760	1.09 (0.81-1.46), *P* = .589
**Secondary analysis of thyroid status in the second/third trimester**	1616 (100)	303 (18.8)		
Taking antithyroid drugs	291 (18.0)	19 (6.5)	ref	ref
Prior definitive treatment	360 (22.3)	142 (39.4)	9.29 (5.58-15.48), *P* < .001	9.16 (5.46-15.36), *P* < .001
No prior definitive treatment and not taking antithyroid drugs	965 (59.7)	142 (14.7)	2.46 (1.50-4.05), *P* < .001	2.50 (1.51-4.13), *P* < .001

Adjusted models includes maternal age, year of pregnancy start, multiple pregnancy, index of multiple deprivation, smoking status, pre-existing diabetes, pre-existing hypertension. Further adjustment for BMI did not materially alter the model estimates (see supplementary Table S4 (26).

Crude and adjusted models exclude 1 pregnancy with missing smoking status.

Abbreviations: OR, odds ratio.

**Figure 4.**
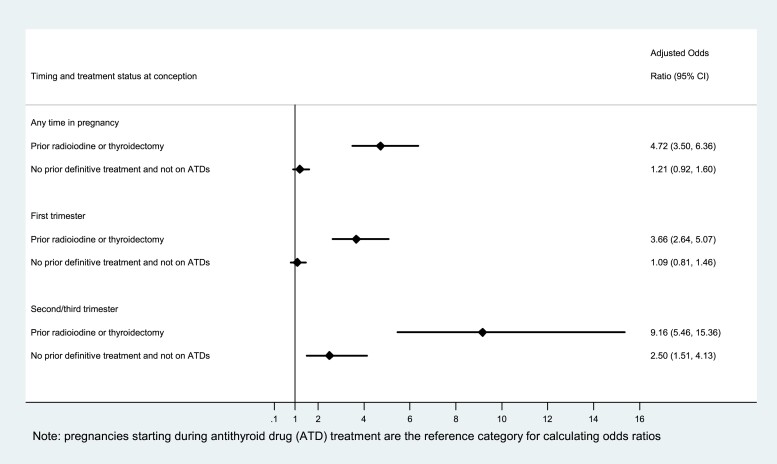
Suboptimal thyroid status in pregnancy by prior treatment.

In an unadjusted model, pregnancies with prior radioiodine or thyroid surgery had markedly increased odds of having suboptimal thyroid status compared with pregnancies starting during antithyroid drug treatment (crude OR 4.48, 95% CI 3.31-6.06). The magnitude of this association increased further following adjustment for maternal age, year of pregnancy start, multiple pregnancy, IMD, smoking status, pre-existing diabetes, and pre-existing hypertension (adjusted OR 4.72, 95% CI 3.50-6.36) (Table 5 and Fig. 4). Further adjustment for BMI in a model restricting to pregnancies with BMI data (n = 2370) did not materially alter the model estimates (adjusted OR 4.54, 95% CI 3.35-6.17) (Table S4 (26)). No difference in odds of suboptimal thyroid status was observed between those who had not undergone thyroid surgery or radioiodine and were not on antithyroid drugs at conception and pregnancies starting during antithyroid drug treatment (adjusted OR 1.21, 95% CI 0.92-1.60) (Table 5 and Fig. 4).

Our secondary analysis of thyroid status during the first trimester (n = 1858 pregnancies with a first trimester TSH record) showed a similarly marked increase in odds of suboptimal thyroid status among pregnancies with prior radioiodine or thyroid surgery compared with pregnancies starting during antithyroid drug treatment (adjusted OR 3.66, 95% CI 2.64-5.07) (Table 5 and Fig. 4). An even larger increase in odds of suboptimal thyroid status was observed during the second/third trimester (n = 1616 pregnancies with a second or third trimester TSH record) among pregnancies with prior radioiodine or thyroid surgery (adjusted OR 9.16, 95% CI 5.46-15.36) (Table 5 and Fig. 4). The magnitude and strength of these associations persisted after further adjustment for BMI (first trimester adjusted OR 3.60, 95% CI 2.57-5.03; second/third trimester adjusted OR 9.05, 95% CI 5.29-15.47).

Comparing pregnancies with no prior radioiodine or thyroid surgery and not on antithyroid drugs at conception with pregnancies starting during antithyroid drug treatment, we observed no difference in odds of suboptimal thyroid status during the first trimester (adjusted OR 1.09, 95% CI 0.81-1.46) (Table 5 and Fig. 4); however, in the second/third trimester, the odds of suboptimal thyroid status were higher among women who had not undergone thyroid surgery or radioiodine and were not on antithyroid drugs at conception (adjusted OR 2.50, 95% CI 1.51-4.13) (Table 5 and Fig. 4).

Our post hoc analysis of pregnancies with prior radioiodine or thyroid surgery showed that 90.6% (n = 492) were prescribed levothyroxine after treatment, of which 264 (53.7%) had suboptimal thyroid status vs 35.3% (n = 18) of those not prescribed levothyroxine after treatment. Our analysis of preconception TSH levels identified 1013 pregnancies with a TSH record in the 90 days before conception (40.5% of the analysis cohort), of which 284 (28.0%) had preconception TSH >2.5 mU/L. Women with prior radioiodine or thyroid surgery were more likely to have TSH >2.5 mU/L before conception (n = 94, 45.2%) than women taking antithyroid drugs at conception (n = 47, 16.6%) and women with no prior radioiodine or thyroid surgery and not taking antithyroid drugs at conception (n = 143, 27.4%) (Table S5 26)). Among 158 pregnancies with prior radioiodine or thyroid surgery and a TSH record in the first trimester and <90 days before conception, 77 (48.7%) had suboptimal thyroid status during the first trimester, with the highest risk seen among the subgroup with TSH >2.5 mU/L before conception (n = 42, 57.5%) compared with pregnancies with preconception TSH ≤2.5 mU/L (n = 35, 41.2%) (Table S6 (26)).

### Sensitivity Analyses

Our analysis of 1435 pregnancies with both TSH and FT4 data yielded similar findings to the main analysis (adjusted OR 4.25, 95% CI 2.97-6.10) comparing pregnancies with prior radioiodine or thyroid surgery with pregnancies starting during antithyroid drug treatment (Table S7 (26)). We observed an even larger increase in odds of suboptimal thyroid status among pregnancies with prior radioiodine or thyroid surgery in our analysis of 1529 pregnancies eligible for HES linkage (adjusted OR 5.32, 95% CI 3.61-7.83) (Table S8 (26). Our analysis using a lower TSH threshold of 2.5 mU/L classified 1003 pregnancies (40.1%) with suboptimal thyroid status, of which 871 (86.8%) were due to elevated TSH, and 132 (13.2%) were due to suppressed TSH plus raised FT4. Consistent with the primary analysis, pregnancies with prior radioiodine or thyroid surgery had markedly increased odds of suboptimal thyroid status compared with pregnancies starting during antithyroid drug treatment (adjusted OR 5.35, 95% CI 4.01-7.14) (Table S9 (26)). A small increase in odds of suboptimal thyroid status was observed among pregnancies with no prior radioiodine or thyroid surgery and not exposed to antithyroid drugs at conception compared with pregnancies starting during antithyroid drug treatment (adjusted OR 1.30, 95% CI 1.02-1.65) (Table S9 (26)).

## Discussion

Our results from a large UK CPRD cohort show that the management of women with a prior diagnosis of hyperthyroidism needs substantial improvement in order to optimize pregnancy outcomes as measured by TSH and FT4 levels. Only 53.1% of women with a history of hyperthyroidism had thyroid monitoring during pregnancy, of which 28.1% had suboptimal thyroid status. This is important to address as maintaining optimal thyroid status in these women during pregnancy is an important modifiable risk for a wide variety of adverse pregnancy outcomes, including fetal loss (1), and may explain the higher rate of obstetric complications in women with a history of hyperthyroidism (32).

Women who had received definitive treatment (radioiodine or surgery) had particularly suboptimal thyroid hormone replacement with a high risk of being outside target (51.9%) (Table 5) and only 65.3% had a TSH measured during pregnancy. Suboptimal management was reflected however in all groups due to infrequent and inadequate thyroid function testing during pregnancy and levels commonly outside the target reference range. The median number of thyroid function tests during pregnancy was 2 (IQR 1-3), which is far lower than American Thyroid Association guidance of thyroid function tests every 4 to 6 weeks in those on antithyroid drugs (16) and is inadequate for those on levothyroxine (16). This strongly suggests there is likely to be a combination of reflexive rather than predictive dose titration of levothyroxine exacerbated by irregular thyroid function monitoring. Indeed, women who had previously had definitive treatment were more likely to have suboptimal thyroid status than pregnancies starting during antithyroid drug treatment (adjusted OR 4.72, 95% CI 3.50-6.36), with an even larger difference seen between these 2 groups during the latter two-thirds of pregnancy (adjusted OR 9.16, 95% CI 5.46-15.36). This may reflect deterioration in thyroid control being detected after increased thyroxine demands after 12 weeks of pregnancy.

This finding is also in keeping with our previous work in a CPRD cohort which has shown that women with established hypothyroidism have poor thyroid control in pregnancy (21). This needs addressing as poor control has been associated with increased odds of fetal loss (21, 33). Although the subgroup of patients who received definitive treatment are more likely to be known to endocrinology teams, they are also more likely to have complete thyroid failure than those with primary hypothyroidism thereby requiring predictive dosage adjustments such as doubling the levothyroxine dose on 2 days of the week at confirmation of pregnancy (34). Taken together this highlights that endocrinologists need to warn patients about the importance of maintaining optimal thyroid status during pregnancy in those who receive definitive treatment and require levothyroxine, and this should form a key component of the consent process for definitive treatment and in prepregnancy counseling for women with hyperthyroidism. It is particularly striking that many women (28.0%), particularly those with prior definitive treatment (45.2%), had suboptimal TSH levels prior to pregnancy, thus substantially increasing the risk of suboptimal TSH occurring in pregnancy when demand for thyroxine is increased.

The temporal trends we have observed are also intriguing with definitive treatment falling steadily between 2000 and 2017 despite growing awareness of the adverse consequences of antithyroid drug exposure during pregnancy (1, 3). This may reflect growing confidence in the management of thyroid disease in pregnancy. They also show how PTU use has slightly declined, which is at odds with current recommendations (3) which has encouraged PTU in the first trimester. We also observed that switching antithyroid drugs remained a relatively uncommon practice of 3.3% of pregnancies overall (Fig. 3) but 19.8% of first trimester antithyroid drug exposed pregnancies. As expected, due to CBZ being the treatment of choice in treating hyperthyroidism outside pregnancy and an increased risk of birth defects with exposure to CBZ in early pregnancy, we observed that switching antithyroid drugs was more common for CBZ to PTU (32.6% of first trimester CBZ-exposed pregnancies) than from PTU to CBZ (6.0% of first trimester PTU-exposed pregnancies) (Table 1). The continued use of CBZ in women becoming pregnant could reflect suboptimal prenatal counseling in women of childbearing age, so that they become pregnant when established on CBZ.

The paucity of thyroid function testing during pregnancy needs addressing. Surprisingly 26.4% of patients on antithyroid drugs during pregnancy did not have thyroid function recorded during the pregnancy. Thyroid function in women on antithyroid drugs needs close monitoring as over- or undertreatment may worsen pregnancy outcomes. Furthermore many women are able to stop antithyroid drugs in the second trimester and overtreatment may result in substantial risk of fetal hypothyroidism (35). The lack of regular and repeated thyroid function testing in our dataset is surprising and may reflect a combination of factors, including (1) late presentation of pregnant women, (2) lack of awareness of guidelines from general physicians and midwives, (3) delays in accessing specialty clinics, and (4) incomplete capture of thyroid function tests in our dataset.

Strengths of this study include a large national primary care dataset and pregnancy register, with linked hospitalization data to increase ascertainment of thyroid surgery procedures and radioiodine therapy, and the ability to assess the temporal trends as the guidance and evidence base has changed. However, there are several limitations. The use of read codes for our diagnosis of hyperthyroidism may be subject to misclassification given the variety of codes used (Table S1 (26)). In particular, pregnancies with no prior radioiodine or thyroid surgery and not taking antithyroid drugs at conception may have included some women with transient abnormal thyroid function, which may have resulted in overestimating the prevalence of suboptimal thyroid control among this group. An additional limitation is that the timing of pregnancy start, end, and trimesters are estimated by an algorithm and therefore may be inexact. However, any imprecision is likely to be nondifferential with respect to thyroid treatment and control. Furthermore, we may miss some thyroid function tests measured during pregnancy undertaken in the private sector and we cannot exclude that some of the thyroid function tests performed in specialty care or secondary care were not captured in our dataset. There is also scope for under-ascertainment of treatment. For instance, prescriptions issued outside of general practice and procedures done in a private clinic setting may not be captured, and data on drug adherence are lacking. The criteria we set for abnormal thyroid function is outside the guidelines (16) as we defined suboptimal thyroid status as (1) TSH >4.0 mU/L, or (2) TSH <0.1 mU/L in the presence of raised FT4 (above the reference range). In our primary analysis, we used TSH alone for classification of suboptimal thyroid status in the absence of FT4 data, which may have led to some misclassification of thyroid status, particularly among women taking antithyroid drugs. However, our sensitivity analysis in pregnancies with both TSH and FT4 data showed similar results.

In conclusion, from a large UK cohort we have established that the management of women with pre-existing hyperthyroidism who become pregnant is suboptimal and needs urgent improvement. Women who had preconception definitive treatment (thyroidectomy or radioiodine) are particularly at risk of suboptimal thyroid status in subsequent pregnancy. Addressing the paucity of thyroid function testing and what appears to be inadequate prenatal counseling (avoiding getting pregnant on CBZ/ensuring an early increase in levothyroxine dose in those who have received definitive treatment) will enable thyroid status to be much more optimally managed which should in turn substantially improve pregnancy outcomes.

## Data Availability

Data may be obtained from a third party and are not publicly available. The data used for this study were obtained from the Clinical Practice Research Datalink (CPRD). All data are available via an application to CPRD's Research Data Governance (RDG) Process (see https://www.cprd.com/research-applications). Data acquisition is associated with a fee and subject to ethics approval.

## Abbreviations

BMI: body mass index
CBZ: carbimazole
CPRD: Clinical Practice Research Datalink
FT4: free thyroxine
HES: Hospital Episode Statistics
IMD: Index of Multiple Deprivation
MMI: methimazole
PTU: propylthiouracil
TSH: thyrotropin
